# Depressive Symptoms Are Associated with Analgesic Use in People with Alzheimer’s Disease: Kuopio ALSOVA Study

**DOI:** 10.1371/journal.pone.0117926

**Published:** 2015-02-17

**Authors:** Julia Fiona-Maree Gilmartin, Saku Väätäinen, Soili Törmälehto, J. Simon Bell, Eija Lönnroos, Lotta Salo, Ilona Hallikainen, Janne Martikainen, Anne M. Koivisto, the ALSOVA Study Group

**Affiliations:** 1 Institute of Clinical Medicine, Neurology, School of Medicine, University of Eastern Finland, Kuopio, Finland; 2 Centre for Medicine Use and Safety, Monash University, Parkville, Victoria, Australia; 3 University College London School of Pharmacy, London, United Kingdom; 4 Pharmacoeconomics and Outcomes Research Unit (PHORU), School of Pharmacy, University of Eastern Finland, Kuopio, Finland; 5 Kuopio Research Centre of Geriatric Care, School of Pharmacy, Faculty of Health Sciences, University of Eastern Finland, Kuopio, Finland; 6 Institute of Public Health and Clinical Nutrition, Department of Geriatrics, University of Eastern Finland, Kuopio, Finland; 7 School of Educational Sciences and Psychology, University of Eastern Finland, Kuopio, Finland; 8 Neurology of NeuroCentre, Kuopio University Hospital, Kuopio, Finland

## Abstract

Neuropsychiatric symptoms of Alzheimer’s disease (AD) such as depression may be associated with pain, which according to the literature may be inadequately recognized and managed in this population. This study aimed to identify the factors associated with analgesic use in persons with AD; in particular, how AD severity, functional status, neuropsychiatric symptoms of AD, co-morbidities and somatic symptoms are associated with analgesic use. 236 community-dwelling persons with very mild or mild AD at baseline, and their caregivers, were interviewed over five years as part of the prospective ALSOVA study. Generalized Estimating Equations (GEEs) were used to estimate unadjusted and adjusted odds ratios (ORs) for the factors associated with analgesic use over a five year follow-up. The proportion of persons with AD using any analgesic was low (13.6%) at baseline and remained relatively constant during the follow-up (15.3% at Year 5). Over time, the most prevalent analgesic changed from non-steroidal anti-inflammatories (8.1% of persons with AD at Year 1) to acetaminophen (11.1% at Year 5). Depressive symptoms (measured by the Beck Depression Inventory, BDI) were independently associated with analgesic use, after effects of age, gender, education, AD severity, comorbidities and somatic symptoms were taken into account. For every one unit increase in BDI, the odds of analgesic use increased by 4% (OR = 1.04, 95% confidence interval CI = 1.02-1.07). Caregiver depressive symptoms were not statistically significantly associated with analgesic use of the person with AD. Depressive symptoms were significantly associated with analgesic use during the five year follow-up period. Possible explanations warranting investigation are that persons with AD may express depressive symptoms as painful somatic complaints, or untreated pain may cause depressive symptoms. Greater awareness of the association between depressive symptoms and analgesic use may lead to safer and more effective prescribing for these conditions.

## Introduction

Alzheimer’s disease (AD) is characterized by progressive neurodegeneration, impaired cognition, function, and behavioral and social skills. Neuropsychiatric symptoms of AD, such as apathy, depression, irritability and agitation [1], can cause strain to the individual, their caregiver and the healthcare system [2]. Improving quality of life (QoL) is one of the main aims of AD management, which includes treating the symptoms of AD and any concurrent conditions [3]. As persons with AD are often older individuals with multiple co-morbidities, pain relief should be one of the mainstays of therapy [4]. The literature has shown that standardized, stepwise analgesic treatment protocols can improve agitation, aggression and pain in persons with moderate to severe dementia, and have the potential to reduce antipsychotic use [5].

Pain appears to be inadequately recognized and treated in persons with varying stages of AD (including mild AD) and other dementias [4, 6–11]. Although many studies have shown that persons with AD and other dementias use fewer analgesics than those without dementia [6–10], the reverse phenomenon has also been shown [12, 13].[7] Nygaard et al (2005) identified that a lower proportion of older Norwegian care home residents with a dementia diagnosis received analgesics ‘when required’ (12%) compared to those who were cognitively impaired and did not have a dementia diagnosis (27%), or those without cognitive impairment (33%) [10]. Similarly, Morrison et al (2000) reported that persons with advanced dementia who fractured their hip, received one-third of the mean daily dose of opioid analgesia postoperatively compared to those without cognitive impairment [4]. This is despite the fact that persons with dementia are likely to experience pain to a similar extent as those without dementia [4, 11].

Despite these findings, there is a paucity of published literature exploring analgesic use in persons with AD. Gallini et al (2013) outlined that persistent analgesic use in persons with mild to moderate AD has not been examined in the published literature [9], despite the importance of involving these individuals in research. These individuals are often not under nursing home care, their persistent pain may worsen AD progression, and AD progression may influence the experience of pain and subsequent need for analgesics [9]. Prevalence of acute analgesic use (use at any study visit) was found to be 25.6% (152/595) and persistent use (use on at least two consecutive study visits, six months apart) was 13.1% (78/595) [9]. The most common analgesic used alone or in combination was acetaminophen [9]. Females and individuals with osteoarthritis were more likely to use analgesics persistently and statistically significant associations between persistent analgesic use, AD duration and recent change in Mini Mental State Examination (MMSE) were identified [9]. While analgesic use may not be the focus of the following studies [14, 15], literature exploring other treatment regimens in persons with mild to moderate AD can still give an indication of analgesic use in this population. In a study examining the effects of obstructive sleep apnoea treatment on cognitive function, 67.3% (35/52) of individuals were stable on analgesics [14], while in a study evaluating donepezil against placebo it was found that analgesics were used by 32.0% of 2376 participants [15]. The published literature exploring pain perception in persons with mild to moderate AD can also inform this topic [16, 17]. Jensen-Dahm et al (2014) examined pain tolerance in persons with mild to moderate AD and identified that they exhibited a lower mechanical pain tolerance compared to controls [17]. It was also postulated that the reduced verbal report of pain may be a function of impaired communication or memory problems associated with AD [17]. Cole et al (2006) identified that the perception of pain was not diminished in persons with mild to moderate AD and postulated that this pain perception may differ when compared to persons with more advanced stages of AD [16]. These findings support the theory that patterns of analgesic use may differ between those with AD and those without, and among the different stages of AD severity.

There are also limitations associated with the current literature assessing analgesic use in persons with AD. Data concerning both the person with AD and their caregiver are often not collected [4, 8, 9], most studies are cross-sectional rather than longitudinal [7, 10, 11], and medication utilization studies using administrative pharmacy information have not analyzed rich clinical data [6].

The present study aimed to examine the factors associated with analgesic use in persons with AD. In particular, how AD severity, functional status, neuropsychiatric symptoms of AD (e.g. behavioral and depressive symptoms), co-morbidities and somatic symptoms are associated with analgesic use. The purpose of this study was to further understand how analgesics are used in persons with AD.

## Methods

### Data source and study sample

This study utilized data collected as part of the ALSOVA study, a prospective, five year, rehabilitation study, conducted by the Department of Neurology, University of Eastern Finland [3, 18]. The primary aim of the ALSOVA study was to evaluate whether an early psychosocial rehabilitation intervention, combining education and support for persons with very mild (Clinical dementia rating, CDR 0.5) or mild (CDR 1) AD and their caregivers, could postpone institutionalization of the person with AD. The ALSOVA study intervention comprised intensive psychosocial courses conducted during the first two years after AD diagnosis. These courses involved: evaluation of the current family situation, lectures about AD, increasing awareness of available social services and methods for caregivers to cope with stress, and social activities for the caregiver and person with AD [19]. One of the secondary ALSOVA study aims was to generate new knowledge to improve the care of persons with AD, this included describing medication utilization. The ALSOVA study design and sample have been previously described [3, 18, 20].

In the ALSOVA study, persons with AD and their caregivers were recruited from April 2002 to September 2006 from hospital memory polyclinics in three hospital districts of Eastern and Central Finland, during the first year after AD diagnosis [3]. To be eligible for inclusion in the study, persons with AD had to have very mild or mild AD [3]. They had to provide informed consent, live in the community, be free of comorbidities that could affect cognition at baseline, and have a family caregiver (spouse, sibling, child) who they were preferably in daily contact with [3, 20].

A geriatrician or neurologist diagnosed AD using the criteria devised by the National Institute of Neurological and Communicative Disorders and Stroke and AD and Related Disorders Association (NINCDS-ADRDA), and the Diagnostic and Statistical manual of Mental Disorders (DSM-IV) [3, 20]. To diagnose AD, the NINCDS-ADRDA recommend obtaining information from the individual’s medical history, neuropsychological tests, laboratory studies and neurologic, psychiatric and clinical examinations [21]. Probable AD can be diagnosed if there is insidious onset of cognitive deterioration, in the absence of other systemic or brain diseases that could account for progressive memory and other cognitive deficits [21]. Possible AD may be diagnosed in the presence of other comorbidities if AD is considered the more likely cause of the progressive dementia, and definite AD may be diagnosed following histopathologic confirmation [21]. The DSM-IV considers the individual’s family history, evidence of decline in memory and learning and cognition, the absence of comorbidities that are likely to be contributing to the cognitive decline, and the presence of AD genetic mutations [22]. In this study, a diagnosis of very mild AD (prodromal) was confirmed by the study neurologist, using criteria proposed by Dubois et al (2007) [23]. All persons with AD also underwent comprehensive diagnostic evaluation and brain imaging [3]. The mean time between AD diagnosis and participants’ baseline visit was an average of five months [20].

After the persons with AD and their caregivers were recruited, they were followed-up annually for five consecutive years (i.e. data were collected from 2002 to 2011). Each annual study visit included a neuropsychological test battery conducted by a psychologist, and a structured interview between the study nurse, the caregiver and the person with AD [3]. During these visits, socio-demographic details (age, gender, years of education) and general health (comorbidities and medication use) were collected [3].

In this study, the longitudinal data were used to examine factors associated with analgesic use in persons with AD. Although this was not the primary aim of the ALSOVA study, the rigorous medication assessment made the secondary analyzes of analgesic use possible. Published studies that have examined medication use data collected as part of other, larger studies, supports the rationale of this study [3, 9, 18, 20]. The study sample comprised 236 patient-caregiver dyads and up to five years of follow-up data, resulting in a total of 806 person observations (data points). Data from the fourth study visit were omitted from the present analyzes as they were collected using a postal survey, instead of structured interviews.

### Medication assessment and analgesic use

At each study visit interview, caregivers were asked to report all medications (including prescription, non-prescription, complementary and alternative medications) used by the person with AD. Alternative medications particularly referred to non-prescription ‘natural’, herbal or mineral products, such as gingko biloba. Information collected on structured forms included medication start and end dates, regularity of use, and whether medications were ‘still in use’. Medications ‘still in use’ included those that were regularly used during the study, for example, daily or monthly. Medications were categorized using the Anatomical Therapeutic Chemical (ATC) Classification System recommended by the World Health Organization (WHO) [24].

Analgesics were defined as non-steroidal anti-inflammatories (NSAIDs, ATC code: M01A), opioids (N02A), and acetaminophen and high dose acetylsalicylic acid (N02B). Low-dose acetylsalicylic acid, glucosamine and anti-migraine medications were not classified as analgesics. Analgesic use was dichotomized as ‘using analgesics’ or ‘not using analgesics’. In this study, the person with AD was defined as using analgesics at each study visit if they were reported to have analgesic medications ‘still in use’ at the time of the study visit, including medications used in single doses, on an ‘as-needed basis’, or regularly.

### Factors associated with analgesic use (parameters to explore)

Factors potentially associated with analgesic use were first identified by their clinical relevance [25, 26]. The hypothesis outlining what factors may be associated with analgesic use was defined before conducting analyzes. It was hypothesized that AD severity (evaluated using the CDR scale) [27], functional status (evaluated using the AD Cooperative Study Activity of Daily Living inventory, ADCS-ADL) [28], neuropsychiatric symptoms of AD (e.g. behavioral symptoms evaluated using the Neuropsychiatric inventory, NPI [29] and depressive symptoms evaluated using the Beck Depression Inventory, BDI) [30]), co-morbidities and somatic symptoms (e.g. symptoms of discomfort, evaluated using one item from the 15D health-related QoL questionnaire) [31] were associated with analgesic use. Although this study only included persons with very mild or mild AD at baseline, AD severity was still considered a potential factor associated with analgesic use, as the severity of AD in the study population increased over the study follow-up period.

### Tools and instruments

The severity of AD was evaluated using the CDR scale [27] and its Sum of Boxes (CDR-SOB) applicant [32]. The CDR is a widely used and validated instrument used to measure the severity and progression of dementia. The CDR scale includes six domains, with scores in each domain combined to obtain a composite score (no dementia = 0, very mild = 0.5, mild = 1, moderate = 2, and severe dementia = 3) and a sum of boxes score [18] ranging from 0 to 18 [32]. The terms very mild (CDR 0.5) and mild AD (CDR 1) were used in the ALSOVA study to describe the different stages of AD at baseline. Those with CDR 0.5 had amnestic mild cognitive impairment and other biomarkers that supported the diagnosis of AD, as described by criteria proposed by Dubois et al (2007) [23]. O’Bryant et al (2008) identified that CDR-SOB scores compare well with CDR scores used for dementia staging [32]. A CDR score of 0.5 corresponds to a CDR-SOB score of 0.5–4.0 (indicating questionable impairment to very mild dementia) [32]. A CDR score of 1 corresponds to a CDR-SOB score of 4.5–9.0 (indicating mild dementia) [32]. Additionally, the MMSE scores can be used as a surrogate measure for the CDR and can therefore be mapped to correspond to CDR scores, where MMSE 30 = CDR 0, MMSE 26 to 29 = CDR 0.5, MMSE 21 to 25 = CDR 1, MMSE 11 to 20 = CDR 2, and MMSE 0 to 10 = CDR 3 [33].

The existence and degree of behavioral symptoms was evaluated using the 12-item Neuropsychiatric inventory (NPI), which identifies caregiver-reported behavioral and psychological problems during the one month prior to the study visit interview [29]. Depressive symptoms and depression were assessed using the validated, self-administered 21-item Beck Depression Inventory (BDI) [30], which has been used to assess persons with dementia [34] and AD [35]. Although BDI is a self-reported tool used to evaluate depression, it captures the subjective experience of depression and gives an indication of clinical depression, with scores from 0–9 indicating no depression, 10–18 mild depression, 19–29 moderate depression, and 30–63 severe depression [36]. The persons with AD were not diagnosed with clinical depression in this study.

As the ALSOVA study did not use a validated instrument to rate pain, somatic symptoms (symptoms of discomfort) were evaluated for their association with analgesic use, and assessed with one screening question ‘discomfort and symptoms’ from the 15D self-administered, validated, health-related QoL questionnaire [31]. Symptoms such as pain, aches, nausea and itch are included in this item [31]. In its entirety the 15D instrument comprises 15 dimensions: breathing, mental function, speech, vision, mobility, usual activities, vitality, hearing, eating, elimination, sleeping, distress, discomfort and symptoms, sexual activity and depression [31]. Each dimension is divided into five grades of severity [31]. The five grades of severity associated with the ‘discomfort and symptoms’ dimension includes: no, mild, marked, severe, and unbearable physical discomfort or symptoms. For this study, marked, severe and unbearable grades were combined into ‘moderate to unbearable discomfort or symptoms’. The 15D can also be used as a preference-based health-related QoL measure when the dimensions are weighted using population-based preferences to obtain a single index score, leading to a final score of between 0 (dead) and 1 (no problems on any dimension) [31]. However, the preference-weighted measures were not within the scope of this study.

The 23-item ADCS-ADL inventory was used to assess caregiver-evaluated ADL, of the person with AD [28]. All comorbidities, other than rheumatoid arthritis or severe arthrosis, had similar, statistically non-significant associations with the odds of using analgesics. For this reason they were combined into a single summary score, ‘number of other comorbidities’.

### Statistical procedures and analyzes

The data were described using means, ranges, proportions and measures of variance (standard errors (SE) of the mean). The association between potential factors and analgesic use were examined by estimating their impact on the odds of the person with AD using analgesics. This was accomplished by using Generalized Estimating Equations (GEEs) to estimate the unadjusted and adjusted odds ratios (ORs) with 95% confidence intervals (CIs) for associations between independent variables (factors) and analgesic use (dependent variable) [37].

GEEs used in this study can be considered as repeated measures logistic regression, as they are an extension of generalized linear models, specified with a logit link function and binomial distribution function. However, the key difference to conventional logistic regression is that GEEs account for the dependent nature of data points, seen in longitudinal study designs where the observations obtained from each study participant are typically correlated with each other [38, 39]. Previously, GEEs have been used in published longitudinal studies examining factors associated with an increased risk of dementia in persons with congestive heart failure [40], the association between digital literacy and decreased cognitive decline in older adulthood [41], and the temporal relationship between depressive symptoms, function, and cognitive status in persons with AD [42]. In this study, GEEs were employed specifically to address the dependent, correlated nature of observations, as each study participant was examined (observed) repeatedly, up to five times i.e. in total, 1 to 5 observations per individual during the study. As GEEs include all study participants in analyzes, rather than only those who remained in the study, participant attrition is also accounted for and an advantage is gained over conventional binary logistic regression. In the GEEs used in this study, variance (i.e. SE and 95% CIs) was estimated using semi-robust estimation.

Initially, GEEs were used to estimate the unadjusted ORs in the bivariate analyzes, to examine which of the clinically relevant potential factors (described above) also had the potential to be statistically significantly associated with analgesic use. Multivariate models were then created by including all clinically relevant and potentially statistically significant (p<0.2 in bivariate analyzes) factors into the models as independent variables. In the multivariate models, these independent variables are considered to predict the dependent variable (analgesic use). In addition, age, gender and education were included in the model as factors potentially affecting analgesic use. The ORs resulting from these multivariate models are referred to as adjusted ORs, as they represent the effect of an independent variable on the odds of an individual using an analgesic, when the effects of the other independent variables included in the model have been accounted for.

All statistical analyzes were performed using Intercooled Stata version 9 (StataCorp. 2005. Stata Statistical Software: Release 9. College Station, TX: StataCorp LP). The conventional threshold of p <0.05 was used as criteria for rejecting the null hypothesis.

### Ethics statement

The ethics committee of Kuopio University Hospital, the Finnish Supervisory Authority for Welfare and Health, and the Finnish Ministry of Social Affairs and Health approved the ALSOVA project. A consent form was signed by both the caregiver and the person with AD. The caregiver also provided proxy consent on behalf of the person with AD.

## Results

### Participants

Baseline characteristics of all 236 participants and their caregivers are presented in Table 1. The study sample of persons with AD comprised an almost even proportion of females (51.3%) and males, who were mostly older (mean age 75.7 years). The majority of persons with AD were either not likely to be suffering from depression (BDI score 0–9, 50.0%) or were potentially suffering from mild depression (BDI score 10–18, 38.6%) and either reported no (51.7%) or mild symptoms of discomfort (39.8%). Caregivers of persons with AD were mostly female (66.5%), had a mean age of 66.2 years, were a spouse of the person with AD (70.3%) and were either not likely to be suffering from depression (55.5%) or were potentially suffering from mild depression (35.2%). The behavioral disturbances which persons with AD exhibited included apathy, depression, irritability and agitation [3].

**Table 1:** Characteristics of persons with AD and their caregivers at baseline.

Person with AD	n = 236
Female gender, n (%)	121 (51.3)
Age, mean years (range)	75.7 (53.8–90.3)
Education, mean years (range)	7.6 (1.0–20.0)
Depressive symptoms (BDI), mean (range)	10.5 (0–38)
0–9, n (%)	118 (50.0)
10–18, n (%)	91 (38.6)
19–63, n (%)	27 (11.4)
Severity of AD (CDR-SOB), mean (range)	4.1 (1–8)
Global CDR score, n (%)	
0.5	128 (54.2)
1.0	108 (45.8)
2.0	0 (0.0)
3.0	0 (0.0)
Functional status (ADCS-ADL), mean (range)	64.6 (33–78)
Behavioral symptoms (NPI), mean (range)	8.9 (0–50)
Rheumatoid arthritis or severe arthrosis, n (%)	77 (32.6)
Number of other comorbidities, mean (range)	1.7 (0–6)
15D—discomfort and symptom item, n (%)	
None	122 (51.7)
Mild	94 (39.8)
Moderate to unbearable	20 (8.5)
Caregiver	n = 236
Female gender, n (%)	157 (66.5)
Age, mean years (range)	66.2 (35.6–84.4)
Education, mean years (range)	9.3 (4–21)
Relationship to person with AD, n (%)	
Spouse	166 (70.3)
Child	55 (23.3)
Other	15 (6.4)
Depressive symptoms (BDI), mean (range)	9.3 (0–32)
0–9, n (%)	131 (55.5)
10–18, n (%)	83 (35.2)
19–63, n (%)	22 (9.3)

AD = Alzheimer’s disease; BDI = Beck Depression Inventory; CDR = Clinical Dementia Rating; SOB = Sum of boxes.

The proportion of persons with AD who used analgesics was low at baseline (n = 32, 13.6% of all persons with AD) and remained relatively constant over the follow-up period (n = 11, 15.3% at Year 5). At baseline, the majority of persons with AD who were using analgesics, were using NSAIDs (n = 19, 8.1%), while acetaminophen alone became the most prevalent analgesic used at Year 5 (n = 8, 11.1%). Over the same time period, the most commonly used medications per category were ibuprofen (NSAID), acetaminophen (other analgesics and antipyretics), and codeine in combination with acetaminophen (opioids), closely followed by tramadol (opioids). Eight hundred and six observations of any analgesic use were collected over the five years, resulting in 109 observations of analgesic use (Table 2).

**Table 2:** Types of analgesics used by persons with Alzheimer’s disease, n (%).

	Baseline (n = 236)	Year 1 (n = 198)	Year 2 (n = 168)	Year 3 (n = 131)	Year 5 (n = 73)
Any analgesic use	32 (13.6)	21 (10.6)	23 (13.7)	22 (16.8)	11 (15.3)
NSAIDs (M01A)	19 (8.1)	8 (4.0)	13 (7.7)	4 (3.1)	3 (4.2)
Analgesic (N02)	16 (6.8)	13 (6.6)	13 (7.8)	19 (14.5)	9 (12.3)
Other analgesics and antipyretics (N02B)	13 (5.5)	11 (5.6)	9 (5.4)	17 (13.0)	8 (11.1)
Opioids (N02A)	3 (1.3)	3 (1.5)	5 (3.0)	3 (2.3)	1 (1.4)

Table 3 describes the characteristics of persons with AD who used analgesics at different follow-up points. Over the five year follow-up, the proportion of persons reporting no depressive symptoms increased compared with other BDI categories (34.3% to 62.5%), the severity of AD measured by CDR increased (moderate AD 0% to 36.4%, and severe AD 0% to 27.3%), and functional status decreased (mean ADCS-ADL 63.3 to 36.9). Behavioral symptoms measured by mean NPI increased in severity (9.9 to 18.2) and the proportion of persons reporting arthritic conditions decreased (62.5% to 45.5%). Reporting of mild discomfort increased compared with other categories of discomfort (46.9% to 71.4%).

**Table 3:** Characteristics of persons with AD who used analgesics over the five year follow-up.

	Baseline (n = 32)	Year 1 (n = 21)	Year 2 (n = 23)	Year 3 (n = 22)	Year 5 (n = 11)
Female gender, n (%)	23 (71.9)	13 (61.9)	13 (56.5)	12 (54.5)	7 (63.6)
Age, mean years (range)	75.7 (63.2–86.6)	79.5 (70.1–91.3)	79.4 (65.3–87.4)	83.3 (72.4–90.4)	81.0 (68.2–87.6)
Education, mean years (range)	6.8 (1–14)	7.3 (4–14)	6.7 (1–11)	6.2 (1–11)	6.5 (6–10)
Depressive symptoms (BDI), mean (SE)	13.8 (1.4)	12.3 (1.4)	14.1 (1.8)	11.6 (1.8)	9.9 (2.6)
0–9, n (%)	11 (34.3)	9 (42.9)	9 (40.9)	11 (55.0)	5 (62.5)
10–18, n (%)	13(40.6)	9 (42.9)	7 (31.8)	4 (20.0)	2 (25.0)
19–29, n (%)	7 (21.9)	3 (14.3)	5 (22.7)	4 (20.0)	1 (12.5)
30–63, n (%)	1 (3.1)	0 (0.0)	1 (4.5)	1 (5.0)	0 (0.0)
Severity of AD (CDR-SOB), mean (SE)	4.3 (0.3)	6.2 (0.6)	6.9 (0.6)	9.3 (0.8)	10.7 (1.3)
Global CDR score, n (%)					
0.5	16 (50.0)	2 (9.5)	4 (17.4)	1 (4.5)	1 (9.1)
1.0	16 (50.0)	16 (76.2)	12 (52.2)	10 (45.5)	3 (27.3)
2.0	0 (0.0)	3 (14.3)	6 (26.1)	9 (40.9)	4 (36.4)
3.0	0 (0.0)	0 (0.0)	1 (4.3)	2 (9.1)	3 (27.3)
Functional status (ADCS-ADL), mean (SE)	63.3 (1.7)	56.4 (2.9)	48.4 (3.7)	37.4 (4.6)	36.9 (6.5)
Behavioral symptoms (NPI), mean (SE)	9.9 (1.6)	13.0 (2.8)	12.4 (1.8)	15.4 (3.1)	18.2 (7.1)
Rheumatoid arthritis or severe arthrosis, n (%)	20 (62.5)	10 (47.6)	13 (56.5)	12 (54.5)	5 (45.5)
Number of other comorbidities, mean (SE)	1.9 (0.2)	2.2 (0.2)	2.1 (0.2)	2 (0.2)	1.8 (0.5)
15D—discomfort and symptom item, n (%)					
None	11 (34.4)	5 (23.8)	7 (31.8)	7 (35.0)	2 (28.6)
Mild	15 (46.9)	10 (47.6)	12 (54.5)	8 (40.0)	5 (71.4)
Moderate to unbearable	6 (18.8)	6 (28.6)	3 (13.6)	5 (25.0)	0 (0.0)

AD = Alzheimer’s disease; BDI = Beck Depression Inventory; SE = standard error; CDR = Clinical Dementia Rating; SOB = Sum of boxes; ADCS = AD Cooperative Study; ADL = activities of daily living; NPI = Neuropsychiatric Inventory.

Among caregivers of persons with AD who used analgesics, the proportion of caregivers who had depressive symptoms over the five year follow-up increased from 43.8% (n = 14) to 63.6% (n = 7). Over the follow-up, increases were seen in those likely to have mild depression (37.5%, n = 12 at Year 1 and 45.5%, n = 5 at Year 5), and moderate or severe depression (6.3%, n = 2 at Year 1 and 18.2%, n = 2 at Year 5), based on BDI scores.

As illustrated in Table 4, AD severity (CDR-SOB), antidepressant use, or caregiver depressive symptoms (measured by BDI), were not significantly associated with analgesic use in the multivariate analyzes (p>0.1). Depressive symptoms (p = 0.002) and rheumatoid arthritis or severe arthrosis (p = 0.006) were the only factors statistically significantly associated with analgesic use, if symptoms of discomfort were not included in the model (Table 4, Model A). Both depressive symptoms and arthritic conditions were also still significantly associated with analgesic use after symptoms of discomfort were included as an independent variable (Table 4, Model B), even though symptoms of discomfort themselves were significantly associated with analgesic use (Model B, p<0.007). Additionally, when using ADCS-ADL instead of CDR-SOB in the multivariate models (ADCS-ADL and CDR-SOB are highly correlated with each other), similar statistically significant findings for the association between depressive symptoms and analgesic use are produced. The association between ADCS-ADL and analgesic use was not statistically significant. Additionally, no significant association between behavioral symptoms (NPI) and analgesic use was observed in the bivariate analysis and therefore NPI was not included in the multivariate models.

**Table 4:** Generalized Estimating Equation models (multivariate analyzes) exploring the impact of five year patient factors on analgesic use over time (all persons with AD).

Factor	All persons with AD					
	Model A, n = 784			Model B, n = 783		
	OR	95% CI	p	OR	95% CI	p
Depressive symptoms (BDI)	1.04	1.02–1.07	0.002	1.03	1.00–1.06	0.028
Follow-up visit	1.05	0.86–1.29	0.636	0.99	0.81–1.22	0.926
Age, years	1.05	1.00–1.10	0.052	1.05	1.00–1.10	0.035
Female gender	1.54	0.88–2.68	0.131	1.64	0.94–2.87	0.081
Education, years	0.94	0.85–1.04	0.205	0.94	0.85–1.04	0.222
Rheumatoid arthritis or severe arthrosis	2.13	1.24–3.66	0.006	2.13	1.26–3.59	0.005
Number of other comorbidities	1.12	0.91–1.37	0.278	1.15	0.96–1.39	0.137
Severity of AD (CDR-SOB)	0.97	0.88–1.06	0.509	0.98	0.90–1.08	0.749
15D—discomfort and symptom item (vs. none)						
Mild				1.45	0.91–2.29	0.116
Moderate to unbearable				2.73	1.33–5.60	0.006

GEEs with logit link, binomial distribution function and unstructured correlation matrix.

P-value for statistical significance: p<0.05. n represents number of observations included in the model, both individuals who use analgesics and who do not.

AD = Alzheimer’s disease; BDI = Beck Depression Inventory; CDR = Clinical Dementia Rating; SOB = Sum of boxes; GEE = Generalized Estimating Equations

This means that depressive symptoms are independently associated with analgesic use regardless of any other variable included in the models presented in Table 4.

As only a small proportion of persons with AD could be classified as potentially having severe depression in the study sample based on their BDI scores, sensitivity analyzes were conducted with GEEs to exclude these potential outliers (Table 5). When persons with AD who reported severe depressive symptoms (defined as BDI > 29, n = 13) were removed from the multivariate GEE model, the strength of association between depressive symptoms and analgesic use increased. At any data collection point over the follow-up period, only one person with AD who used analgesics had severe depression. Depressive symptoms (p = 0.001) and rheumatoid arthritis or severe arthrosis (p = 0.017) were the only factors statistically significantly associated with analgesic use, if symptoms of discomfort were not included in the model (Table 5, Model A). Both depressive symptoms and arthritic conditions were also still significantly associated with analgesic use after symptoms of discomfort were included as an independent variable (Table 5, Model B), even though symptoms of discomfort themselves were significantly associated with analgesic use (Model B, p = 0.001).

**Table 5:** Generalized Estimating Equation models (multivariate analyzes) exploring the impact of five year patient factors on analgesic use over time (persons with AD and non-severe depression, BDI<30).

Factor	Persons with AD and non-severe depression (BDI<30)					
	Model A, n = 771			Model B, n = 770		
	OR	95% CI	p	OR	95% CI	p
Depressive symptoms (BDI)	1.06	1.02–1.09	0.001	1.04	1.01–1.08	0.018
Follow-up visit	1.08	0.88–1.32	0.480	1.01	0.82–1.24	0.929
Age, years	1.04	0.99–1.09	0.089	1.05	1.00–1.10	0.054
Female gender	1.59	0.90–2.81	0.113	1.73	0.97–3.08	0.063
Education, years	0.94	0.84–1.04	0.196	0.94	0.84–1.04	0.212
Rheumatoid arthritis or severe arthrosis	1.97	1.13–3.44	0.017	1.99	1.15–3.43	0.014
Number of other comorbidities	1.15	0.94–1.39	0.167	1.19	0.99–1.43	0.071
Severity of AD (CDR-SOB)	0.97	0.88–1.06	0.485	0.98	0.89–1.08	0.751
15D—discomfort and symptom item (vs. none)						
Mild				1.42	0.91–2.21	0.126
Moderate to unbearable				3.11	1.55–6.27	0.001

GEEs with logit link, binomial distribution function and unstructured correlation matrix.

P-value for statistical significance: p<0.05. n represents number of observations included in the model, both individuals who use analgesics and who do not.

AD = Alzheimer’s disease; BDI = Beck Depression Inventory; CDR = Clinical Dementia Rating; SOB = Sum of boxes; GEE = Generalized Estimating Equations.

Depending on the statistical model, the OR for depressive symptoms was between 1.03 and 1.06, that is, for every one unit increase in BDI, the odds of analgesic use increases by approximately 3% to 6%.

**Fig 1:**
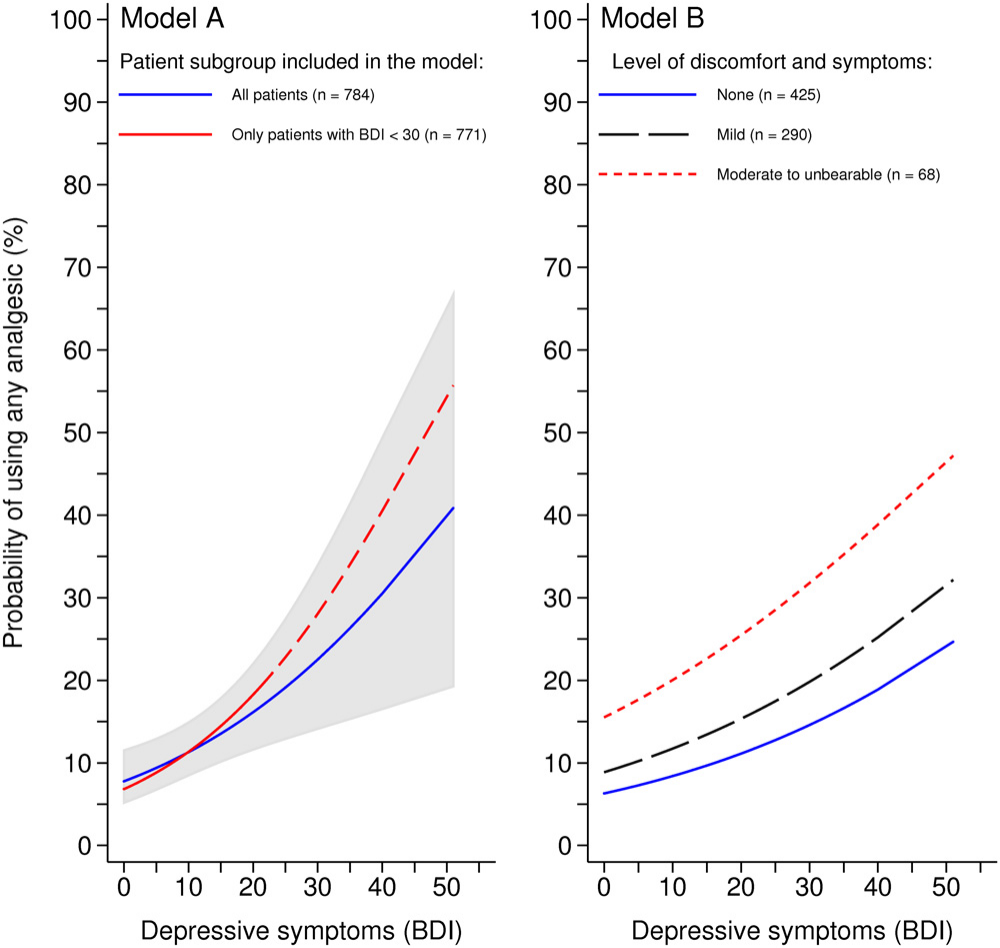
Adjusted probability of analgesic use in relation to depressive symptoms and level of discomfort (means and CIs) in persons with AD. Data is presented for persons with AD. Probability was estimated using GEEs with logit link function, binomial distribution and unstructured correlation matrix. Data is adjusted for age, gender, follow-up visit, years of education, CDR-SOB and comorbidities (arthritic conditions and number of other comorbidities). A BDI score ≥30 was considered to be indicative of severe depression. n represents number of observations included in the model, both individuals who use analgesics and who do not.

Fig. 1 graphically illustrates the association between depressive symptoms and probability of analgesic use (Model A), as well as how symptoms of discomfort affect this relationship (Model B). The rapid increase in the probability of analgesic use as depressive symptoms worsen is apparent even after the data are adjusted for age, gender, education, AD severity and comorbidities. Although symptoms of discomfort were significantly correlated with depressive symptoms (Spearman’s rho = 0.316, p<0.001), no significant interaction between symptoms of discomfort and depressive symptoms was observed in the multivariate GEE models.

## Discussion

The present study examined the factors associated with analgesic use in persons with AD, in particular, how AD severity, functional status, neuropsychiatric symptoms of AD (e.g. behavioral and depressive symptoms), comorbidities and somatic symptoms affect the probability of the person with AD using analgesics. The main finding of this study was that depressive symptoms were independently associated with analgesic use over a five year follow-up of persons with early stage AD at baseline. Furthermore, a significant association was not observed between AD severity, functional status or behavioral symptoms and analgesic use. Although the relationship between pain and depression in persons with dementia has been partly explored in earlier cross-sectional studies [43, 44], there is a paucity of literature exploring the association of analgesic use and depression in AD [6–10]. To the best of our knowledge, this study is the first to report this association and therefore contributes to the growing body of literature concerning analgesic medication management in AD.

There are several potential explanations for the main study finding. Firstly, it is possible that depression and pain were sufficiently coexistent among the study sample to explain the association. The study sample may have been experiencing pain that warranted analgesic use. The association between pain and depression in persons with dementia has been documented [43–45]. Arthritis is also highly prevalent in older Finns [46] and is associated with analgesic use [9]. This study identified that arthritis was significantly associated with analgesic use (p<0.02, OR = 1.97–2.13), along with symptoms of discomfort (p<0.007, OR = 2.73–3.11). Furthermore, a significant correlation between symptoms of discomfort and depressive symptoms was observed. However, although pain could not be evaluated in the study sample, the data showed a significant association between depressive symptoms and analgesic use even after the effect of significant comorbidities (e.g. arthritis and severe arthrosis) and symptoms of discomfort had been adjusted for.

Another possible explanation for the main finding of this study may be that persons with AD use somatic complaints, such as headache or stomach ache, to express depressive symptoms, which in turn leads to analgesic use. Although further research is needed to support this theory, this claim is partially supported by the study conducted by Engedal et al (2011), where it was speculated that ‘multiple physical complaints’ was an unspecific mood symptom expressed by depressed persons with AD [47]. After interviewing 112 persons with AD it was identified that those diagnosed with depression were significantly more likely to present with ‘multiple physical complaints’ than those not diagnosed with depression (p = 0.001) [47]. Even after adjusting for impairment in ADLs and the number of physical disorders, ‘multiple physical complaints’ was significantly associated with depression [47].

It can be suggested that there is the potential for analgesics to be used unnecessarily in persons with AD, if analgesics are treating somatic complaints of depressive symptoms. Further research is needed to confirm this. If this explanation were true, it could lead to polypharmacy [48, 49] and inadequately treated depression. In persons with AD, depression can lead to worse QoL, greater disability in ADL, faster cognitive decline, relatively higher mortality, and a higher frequency of caregiver depression and burden [50–52]. Symptoms of depression occur commonly in persons with AD, but their prevalence can decrease over time [42]. Of persons diagnosed with probable AD in a North American study, Holtzer et al (2005) described a 40% baseline prevalence of depressive symptoms, decreasing to 24% after five years of follow-up [42]. Andreasen et al (2013) identified a 12.4% prevalence of depression in 1612 older persons with dementia from eight different countries [53]. Analgesics, such as opioids, are also associated with adverse drug events including respiratory depression, nausea and vomiting, gastrointestinal reflux and constipation [54], which could in turn lead to increased utilization of health care services.

Despite the potential influence that caregiver depression may have on the assessment of patient depression [50], this study did not show any significant relationship between caregiver depression and analgesic use in persons with AD. At each study visit interview, caregivers were asked to report all medications used by the person with AD. It is possible that interview data used in the current study may not have sufficiently captured the use of over-the-counter analgesics, which caregivers may have more control over. Caregivers may have been more likely to report prescription medications, however, they were instructed to report all medications, and there was no evidence of any systematic underreporting of over-the-counter medications.

Low analgesic use in individuals with dementia [6–10], and no significant change in persistent analgesic use over time [9] has also been identified in the literature. Participants in this study were recruited in the early stages of AD and may not have been experiencing advanced stages of painful comorbidities, which may be more likely to accompany more advanced age and stages of AD, potentially explaining their low overall analgesic use. Participant drop-out may also be an alternative explanation as persons who used more analgesics may have left the study. The most common reasons for participant drop-out included institutionalization (34/236), health deterioration (15/236) or death (20/236). A significant difference in the baseline severity of AD compared to participants who continued through the follow-up was not observed (p = 0.155). Additionally, systematic or major differences between individuals who dropped-out or remained in the study were not observed. Similar drop-out rates have been observed in other studies involving persons with AD [55]. It has been suggested in the literature that low analgesic use in persons with AD and other dementias may be explained by their difficulty in communicating pain, recollecting painful episodes, or requesting analgesics [4]. Additionally, the concern for analgesic adverse drug events [4, 7] and its influence on prescribing patterns, may explain the finding that acetaminophen was the most commonly used analgesic as AD progressed [9, 10], as opposed to NSAIDs.

### Strengths and limitations

Firstly, it is a strength that data analyzed in this study were derived from interviews rather than administrative pharmacy data or prescription registries. Although interviews rely on the accuracy of patient and caregiver self-reports, they provide an opportunity to capture rich clinical data and determine which medications are in current use. However, analgesic doses used were not investigated. Secondly, despite the absence of a statistically significant relationship between caregiver depression and analgesic use, this study provided a unique comparison of linked patient and caregiver data. Thirdly, the repeated and longitudinal process of data collection provided a novel examination of analgesic use over a five year follow-up, compared to studies assessing only cross-sectional data. Lastly, the use of GEEs takes advantage of the longitudinal data by including all participants in analyzes, rather than only those remaining in the study at each time point of data collection.

It is a potential limitation that data were collected in only three Finnish hospital districts. For this reason it is not clear to what extent the findings are generalizable to other regions or countries. However, the patterns of analgesic use identified in this study, are similar to those identified in studies conducted in other countries [6–10]. Secondly, although an AD-specific pain scale was not used, ‘discomfort and symptoms’ was assessed using one part of a validated QoL instrument (15D). Thirdly, medication use between each yearly study visit interview may not have been adequately captured. Fourthly, any relationship between the depressive component of the NPI and analgesic use was not examined in this study. Fifthly, only a few persons with AD potentially had severe depression, however additional analyzes were conducted where these individuals were omitted and an even stronger association between depressive symptoms and analgesic use was identified. Finally, it could not be determined whether there was a cause and effect relationship between depressive symptoms and analgesic use in persons with AD in this study, because data concerning when analgesics were first commenced and when depressive symptoms were first reported were not analyzed. However, an association between depressive symptoms and analgesic use in this study sample was found.

### Future directions

Relative to the number of studies assessing diagnosis and treatment of depression [56], there is limited published research investigating the ways in which persons with AD express their depressive symptoms [47]. This study highlights the importance of considering the different presentations of depression in this setting [57], and the need for further research in persons with AD. More research and quality improvement programs regarding pain assessment and management in dementia are also needed [10, 11, 58].

This study enables greater understanding of how analgesics are used in persons with AD and highlights that pain management is an area that warrants further consideration upon initiation and review of overall AD treatment plans. Further research is needed to explore the association between depressive symptoms and analgesic use and identify whether depressive symptoms lead to greater analgesic use, or whether pain, and consequently analgesic use, leads to greater reporting of depressive symptoms.

## Conclusion

Depressive symptoms in persons with AD were significantly associated with analgesic use during the five year follow-up period. Possible explanations that warrant further investigation is that persons with AD may express depressive symptoms as painful somatic complaints, leading to analgesic use, or inadequately treated pain may cause depressive symptoms. Greater awareness of the association between depressive symptoms and analgesic use may lead to safer and more effective prescribing for these conditions in early stage AD and ultimately improved QoL.
